# Quercetin Alleviates Pulmonary Fibrosis in Silicotic Mice by Inhibiting Macrophage Transition and TGF-β-Smad2/3 Pathway

**DOI:** 10.3390/cimb45040202

**Published:** 2023-04-05

**Authors:** Fei Geng, Lan Zhao, Yuhao Cai, Ying Zhao, Fuyu Jin, Yaqian Li, Tian Li, Xinyu Yang, Shifeng Li, Xuemin Gao, Wenchen Cai, Na Mao, Ying Sun, Hong Xu, Zhongqiu Wei, Fang Yang

**Affiliations:** 1 School of Public Health, Hebei Key Laboratory for Organ Fibrosis Research, North China University of Science and Technology, Tangshan 063000, China; 2 School of Basic Medical Sciences, Hebei Key Laboratory for Chronic Diseases, North China University of Science and Technology, Tangshan 063210, China

**Keywords:** quercetin, macrophage-to-myofibroblast transition, silicosis

## Abstract

Silicosis is a pulmonary disease caused by the inhalation of silica. There is a lack of early and effective prevention, diagnosis, and treatment methods, and addressing silicotic fibrosis is crucial. Quercetin, a flavonoid with anti-carcinogenic, anti-inflammatory, and antiviral properties, is known to have a suppressive effect on fibrosis. The present study aimed to determine the therapeutic effect of quercetin on silicotic mice and macrophage polarity. We found that quercetin suppressed silicosis in mice. It was observed that SiO_2_ activated macrophage polarity and the macrophage-to-myofibroblast transition (MMT) by transforming the growth factor-β (TGF-β)-Smad2/3 signaling pathway in silicotic mice and MH-S cells. Quercetin also attenuated the MMT and the TGF-β-Smad2/3 signaling pathway in vivo and in vitro. The present study demonstrated that quercetin is a potential therapeutic agent for silicosis, which acts by regulating macrophage polarity and the MMT through the TGF-β-Smad2/3 signaling pathway.

## 1. Introduction

Silicosis is a lung disease caused by exposure to large amounts of respirable crystalline silica [1]. The pathology of silicosis is characterized by severe alveolitis, silicotic nodules, and collagen (Col) deposits. To date, no effective early prevention, diagnosis, and timely drug treatment methods have been available [2]. Quercetin is a polyhydroxylated flavonoid with many unique biological properties, including anti-carcinogenic, anti-inflammatory, and antiviral activities. Quercetin is a 3,3′,4′,5,7-pentahydroxy flavonol and a plant flavonoid found in edible fruits and vegetables [3,4,5]. Quercetin also has a suppressive effect on fibrosis [6,7,8,9]. Previous research has revealed that quercetin plays a role in silicosis by inhibiting macrophage senescence, possibly via the senescence-associated secretory phenotype (SASP) [10].

Macrophages in the lungs play an important role in the clearance of pulmonary pathogens and in maintaining steady-state homeostasis [11,12]. The M1 macrophages that overexpress pro-inflammatory cytokines, such as tumour necrosis factor-α (TNF-α), interleukin-6 (IL-6), and inducible nitric oxide synthase (iNOS), are associated with inflammation, antitumoral functions, and graft rejection. The M2 macrophages that overexpress M2 macrophage-associated pro-fibrogenic factors, such as transforming growth factor-β (TGF-β), chitinase 3-like-3 (YM-1), and arginase-1 (Arg), are associated with immune regulation, matrix deposition, and tissue remodeling [13].

Myofibroblasts, which are characterized by the expression of α-smooth muscle actin (α-SMA), have the ability to synthesize collagen and deposit extracellular matrix (ECM) to promote silicosis. Recent studies have shown that macrophages derived from bone marrow cells can differentiate into α-SMA+ myofibroblasts within the injured kidney in a process termed the macrophage-to-myofibroblast transition (MMT) [14]. Cell lineage tracing studies by the adoptive transfer of GFP+ or dye-labelled macrophages (F4/80+) have identified that monocytes/macrophages from bone marrow can give rise to myofibroblasts in a mouse model of unilateral ureteric obstruction (UUO), accounting for more than 60% of α-SMA+ myofibroblasts. Furthermore, many Col I-producing cells isolated from the UUO kidney were bone-marrow-derived GFP + F4/80+ α-SMA+ cells with a predominant CD206+ M2 phenotype. The MMT process was regulated by TGF-β/Smad3 signaling, as the deletion of Smad3 in the bone marrow compartment of GFP+ chimeric mice and Smad3 null bone marrow macrophages in vitro [15]. The majority of MMT cells co-expressed macrophage (CD68 or CD206) and myofibroblast (a-SMA) markers in human and experimental renal allograft rejection. Fate-mapping in Lyz2-Cre/Rosa26-Tomato mice showed that approximately half of a-SMA+ myofibroblasts in renal allografts originated from the recipient’s bone-marrow-derived macrophages. The knockout of Smad3 protected against interstitial fibrosis in renal allografts and substantially reduced the number of MMT cells [16]. The MMT was associated with lung fibrosis in UUO rats, and this process was attenuated after treatment with eplerenone [17]. It was suggested that the MMT is one of the important mechanisms in myofibroblast formation and fibrosis. Nevertheless, there are few reports about the relationship between the MMT and silicosis. The previous study indicated that the positive expression of α-SMA was also found in macrophages in silicotic nodules and located in the membrane similar to “actin rings” stained by phalloidin, another commonly used marker for myofibroblasts [18]. The lost original phenotype macrophage may be related to the process of the MMT. In this study, we investigated the MMT in silicosis and whether quercetin regulated it.

## 2. Materials and Methods

### 2.1. Animal Models

Eight-week-old male C57BL/6 mice were purchased from Vital River Laboratory Animal Technology, China. The study protocol was approved by the Committee on the Ethics of North China University of Science and Technology (LX2019033) and complied with the US National Institutes of Health Guide for the Care and Use of Laboratory Animals. The mice were randomly divided into three groups (*n* = 5) as follows: (1) the control group, subjected to a tracheal perfusion with 50 µL of 0.9% normal saline; (2) the silicosis group, subjected to a one-off non-invasive intratracheal instillation of silica suspension (100 mg/kg) (s5631; Sigma-Aldrich, St. Louis, MO, USA); and (3) the quercetin group, subjected to an intraperitoneal injection of quercetin (100 mg/kg) (Q4951, Sigma–Aldrich, Shanghai, China) every day for 28 days at the time of silica suspension (100 mg/kg) via a one-off non-invasive intratracheal instillation. The mice were sacrificed and part of the lung tissue was dehydrated and embedded in paraffin, while the remaining lung tissue was stored at −80 °C.

### 2.2. Cell Culture

MH-S (mouse macrophage) cells were purchased from the Chinese Academy of Sciences Cell Library (Shanghai, China) and cultured in F12K media (BOSTER; Pleasanton, CA, USA), containing 10% fetal bovine serum (Bovogen Biologicals; Melbourne, Australia) at 37 °C and in 5% CO_2_. The number of cell passages was about 2–5 generations, the subculturing ratio was 1:3, and the cell density was about 85%. When the cell density was about 80%, the MH-S cells were in serum-free F12K. After 8 h of starvation, the cells were randomly divided into control, quercetin (25 μmol/L), SiO_2_ (50 μg/mL), quercetin+ SiO_2_ (in which quercetin was added 2 h before SiO_2_ stimulation), and LY364947+ SiO_2_ (in which LY364947 was added 2 h before SiO_2_ stimulation) groups.

### 2.3. Non-Invasive Measurement of the Pulmonary Function

The pulmonary functions were assessed in whole-body plethysmograph (WBP) chambers (FinePointe WBP, BUXCO Research Systems, INC, Wilmington, NC, USA) according to the manufacturer’s instructions. The mice were placed into the whole-body plethysmograph chambers. The measurement parameters included an adaptation period (10 min), an atomization period (1 s), a reaction period (5 min), and a recovery period (1 min). The main indexes used included the tidal volume (Tvb), minute volume (Mvb), enhanced pause (Penh), pause (PAU), peak inspiratory flow (PIF), peak expiratory flow (PEF), expiratory flow 50 (EF50), and end-expiratory pause (EEP).

### 2.4. Hematoxylin–Eosin Staining

The lungs of the experimental animals were removed and immersed in a formaldehyde solution. The paraffin-embedded lung tissue was cut into approximately 5 μm-thick sections. The sections were deparaffinized and rehydrated. Hematoxylin and eosin dye (BA4025, Baso Diagnostics Inc., Zhuhai, China) were then added in order to observe the pathological morphology, for 3–5 min and 1–2 min, respectively.

### 2.5. Van Gieson’s Staining

The paraffin-embedded lung tissue sections were covered with an equal proportion of hematoxylin A and hematoxylin B for about 3–5 min. Van Gieson’s dye (BA4084, BaSO Diagnostics Inc., Zhuhai, China) was also added.

### 2.6. Immunohistochemical and Immunocytochemical Staining

After van Gieson’s staining, the tissues were fully deparaffinized and hydrated. The antigens were exposed to high-pressure retrieval in a 0.01 mol/L citrate buffer (pH = 6.0). Endogenous peroxidase activity was quenched with 3% hydrogen peroxide for 15 min at room temperature. After blocking with a 5% bovine serum albumin (BSA), the tissues were incubated overnight at 4 °C with α-SMA (ET1607-43, HUABIO, Hangzhou, China) and p-Smad 2/3 (AP0548, ABclonal, Wuhan, China) at a dilution of 1:200,. After washing three times with phosphate-buffered saline (PBS), the sections were combined with the respective secondary antibodies (PV-6000, ZSGB-BIO, Beijing, China) at 37 °C for 40 min. After three additional washes in PBS, the reaction was visualized with 3,3′-diaminobenzidine (DAB) solution (ZLI-9018, Zhongshan, Beijing, China) (reagent 1:reagent 2 = 1:20). After counterstaining with hematoxylin, the slices were viewed under a light microscope and brown staining indicated positive results.

The MH-S cells were immobilized on glass slides [19]. After being immobilized, the slides were subjected to antigen retrieval in a 0.01 mol/L citrate buffer (pH = 6.0) by microwaving and then quenched with 3% hydrogen peroxide for 15 min at room temperature. After blocking with a 5% BSA, the slides were incubated with the primary antibody α-SMA overnight at 4 °C. After three additional washes in PBS, the sections were combined with the respective secondary antibodies and visualized with a DAB solution. After counterstaining with hematoxylin, the slices were viewed under a light microscope and brown staining indicated positive results.

### 2.7. Immunofluorescent Staining

After immunohistochemical and immunocytochemical staining, the tissues were fully deparaffinized and hydrated. The antigens were exposed to high-pressure retrieval in a 0.01 mol/L citrate buffer (pH = 6.0). After washing three times with PBS, the tissues were incubated overnight with α-SMA/F4/80(RT1212, HUABIO, Hangzhou, China) and α-SMA/CD206 (sc58986, Santa Cruz, Dallas, TX, USA). After rinsing with PBS, the sections were incubated with a donkey anti-mouse TRITC secondary antibody and a donkey anti-rabbit FITC secondary antibody (A16028 and A16018, Novex, Frederick, MD, USA) (1:100) for 40 min. The nuclei were stained with DAPI (8961s; Cell Signaling Technology, Inc., Danvers, MA, USA). The sections were examined under an inverted fluorescence microscope (OLYMPUS IX51).

### 2.8. Western Blot

The total protein was extracted from the lungs and MH-S cells by RIPA Lysis Buffer (R0020; Solarbio Life Sciences, Beijing, China) for 20 min on ice and the protein concentration in the extracts was quantified with a BCA (AR1097, BOSTER, Pleasanton, CA, USA) protein assay, according to the manufacturer’s instructions. The proteins were separated via SDS-polyacrylamide gels (10–12%) and electroblotted onto polyvinylidene fluoride (PVDF) membranes. The primary antibodies included Col I (ab34710, Abcam, Cambridge, UK), iNOS (ARG56509, Arigo, Taiwan, China), Arg (ab91279, Abcam, Cambridge, UK), α-SMA (ab5694; Abcam, Cambridge, UK), TGF-β (ARG56429, Arigo, Taiwan, China), TGFβRI (A16983, ABclonal, Wuhan, China), TGFβRII (ARG59501, Arigo, Taiwan, China), p-Smad 2/3, GAPDH (ab181602, Abcam, Cambridge, UK), and β-actin (AC026; ABclonal, Wuhan, China). All of the antibodies were diluted at 1:1000. The PVDF membranes were washed three times with PBST, for 15 min each time. The membranes were further stained by goat anti-rabbit or anti-mouse secondary antibodies (074-1506/074-1806; Kirkegaard and Perry Laboratories, Gaithersburg, MD, USA) at a dilution of 1:5000 in blocking buffer (AR1017, BOSTER, CA, USA). The PVDF membranes were washed three times with PBST for 15 min each time. Immunoblot target bands were visualized using an ECL prime Western blot detection reagent (ZD310A; ZomanBio, Beijing, China). The bands were chemically imaged using the Clinx chemiluminescence imaging system (ChemiScope 6100 EXP; Clinx, Shanghai China). The images were analyzed using ImageJ software (NIH) for gray value, and the results were normalized against the GAPDH or β-actin expression levels and corresponding controls.

### 2.9. Statistical Analysis

Statistical analyses were performed using SPSS 20.0 software (IBM Corp, Armonk, NY, USA) and showed as mean ± SD. Multiple group comparisons were analyzed by One-way ANOVA, whereas two-group comparisons were analyzed using an independent *t*-test. Statistical significance was considered as *p* < 0.05.

## 3. Results

### 3.1. Quercetin Treatment Inproved Lung Functions and Inhibited Collagen Deposition

**Figure 1:**
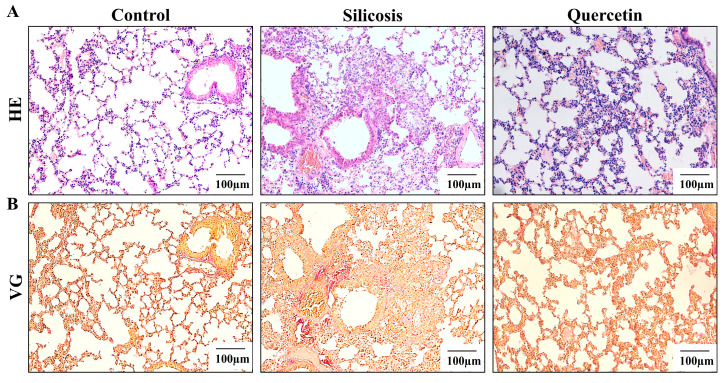
Quercetin inhibited the progression of silicosis in mice. (**A**) Hematoxylin–eosin staining of lung tissues (scale bar = 100 µm). (**B**) Van Gieson’s staining of lung tissues (scale bar = 100 µm).

**Figure 2:**
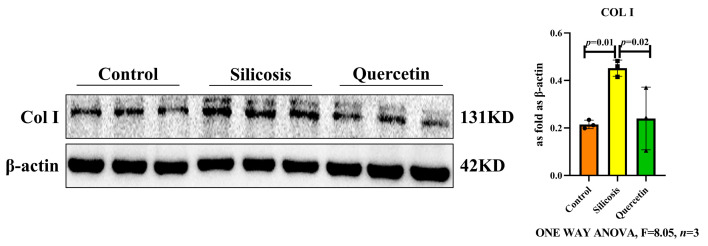
The expression of Col I in the lungs, measured by Western blot. Data are presented as the mean ± SD, *n* = 3 per group.

**Figure 3:**
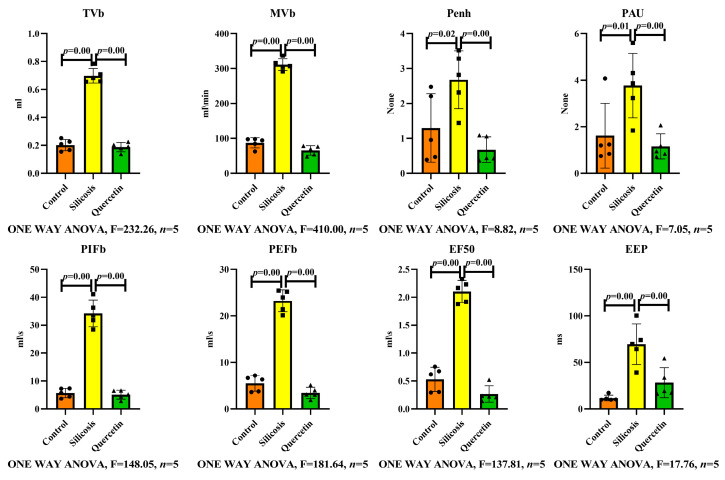
Lung functions of mice, measured by whole-body plethysmograph (WBP) chambers. Data are presented as the mean ± SD, *n* = 5 per group.

The lungs from the silicosis mice models stained with hematoxylin–eosin and van Gieson’s stains showed the formation of a cellular nodule and a Col deposition in the mice exposed to silica for 28 days (Figure 1). The Western blot results showed that the expression of Col I was significantly increased in the silicosis group, the same as the result of van Gieson’s stains (Figure 2). The pathological changes in the lung tissue were significantly alleviated and, in response to silica exposure, the lung functions were regained by quercetin treatment (Figure 3).

Taken together, the results support the claims that SiO_2_ can induce fibrosis and that quercetin treatment can alleviate the structure and function of the lungs. 

### 3.2. Inhibitory Effect of Quercetin on Macrophage Transition in the Lungs of Silicotic Mice

**Figure 4:**
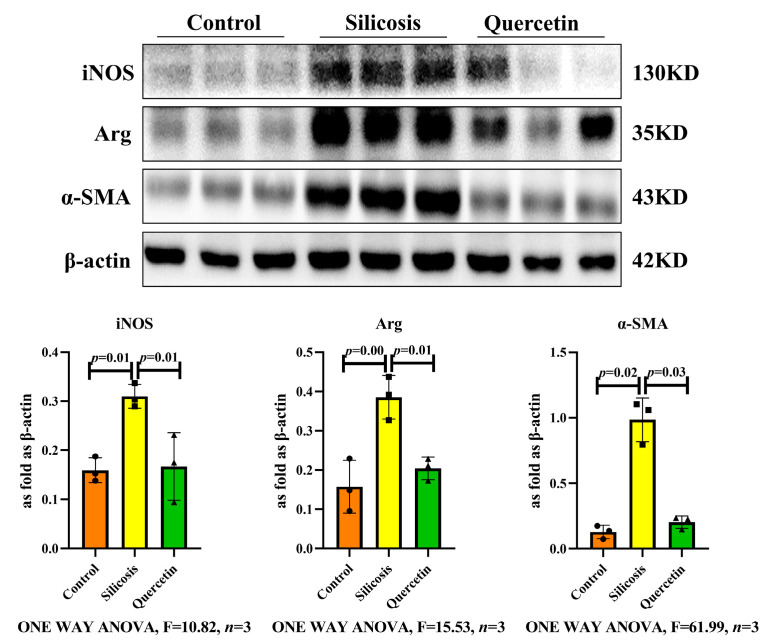
The expressions of iNOS, Arg, and α-SMA in the lungs, measured by Western blot. Data are presented as mean ± SD, *n* = 3 per group.

**Figure 5:**
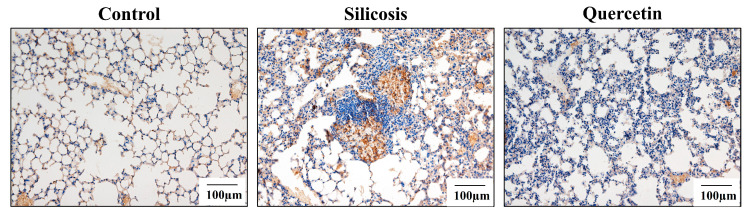
The expression of α-SMA in the lungs, measured by immunohistochemical staining (scale bar = 100 µm).

Macrophages are essential in lung defense, including in the pathogen clearance, immune regulation, and maintenance of homeostasis. Classically activated M1 (pro-inflammatory) and alternatively activated M2 (anti-inflammatory/pro-fibrotic) macrophages play different roles in lung damage, repair, and fibrosis. Western blot results showed that the expressions of iNOS, an M1-specific marker, and Arg, an M2-specific marker, were significantly decreased in the quercetin treatment group compared to in the silicosis group (Figure 4). The activation of α-SMA-positive myofibroblasts plays a key role in the silicosis process. The expression of α-SMA increased in the silicosis group, whereas in the quercetin group, it was suppressed(Figure 5).

**Figure 6:**
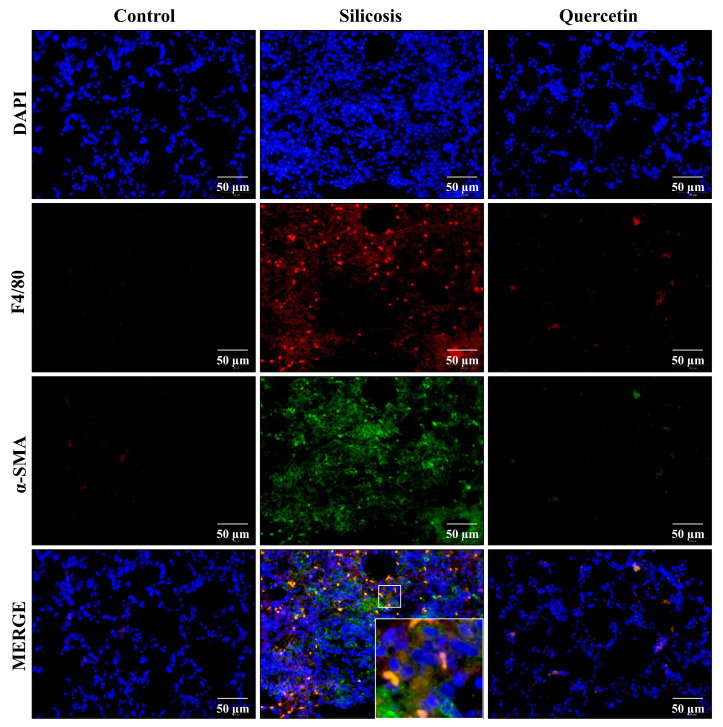
The expressions of F4/80 and α-SMA in the lungs, measured by immunofluorescent staining (scale bar = 50 µm). Blue, DAPI; red, F4/80; green, α-SMA.

**Figure 7:**
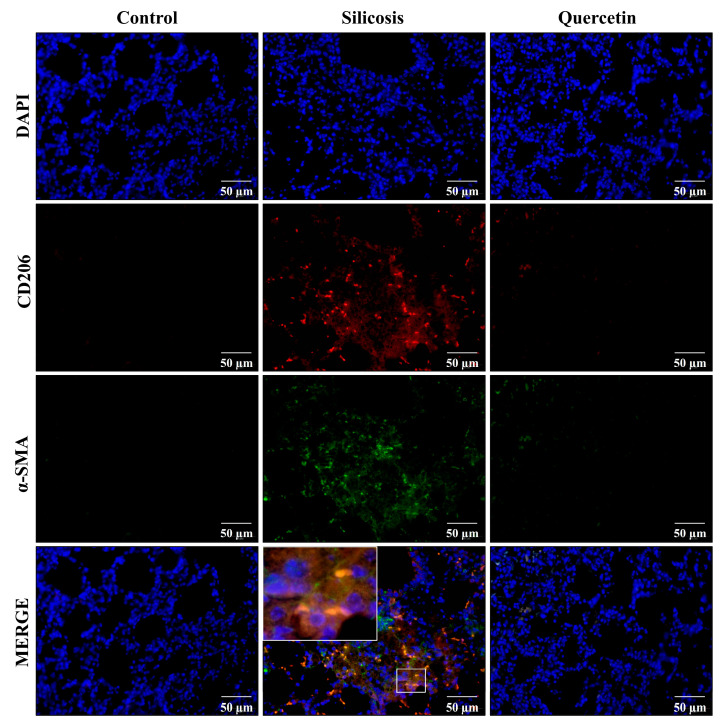
The expressions of CD206 and α-SMA in the lungs, measured by immunofluorescent staining (scale bar = 50 µm). Blue, DAPI; red, CD206; green, α-SMA.

The MMT has been reported as the primary source of myofibroblasts, which are largely derived from M2 macrophages, in vivo models of fibrotic kidney disease [15]. Given the correlation between macrophages and myofibroblasts, we performed a double-staining immunofluorescence analysis using macrophage-specific marker F4/80 and myofibroblast-specific marker α-SMA antibodies. The results showed that the co-expression of F4/80 and α-SMA was significantly higher in the silicosis group, while quercetin could significantly reduce the expression of F4/80/α-SMA (Figure 6). Immunofluorescence staining was performed to identify cells undergoing the MMT based on the co-expression of the M2 biomarker (CD206) and the myofibroblast biomarker (α-SMA). The results showed a massive infiltration of CD206+ macrophages in the lungs of silicotic mice. It is worth noting that the amounts of most of these co-expressed CD206 and α-SMA cells were significantly higher in the silicosis group. However, quercetin treatment significantly reduced the double-positive CD206+/α-SMA+ cells (Figure 7). These findings indicated that the macrophages become polarized and undergoMMT in silicosis, whereas quercetin can reverse the change.

### 3.3. Quercetin Inhibited SiO_2_-Induced Macrophage Transition

**Figure 8:**
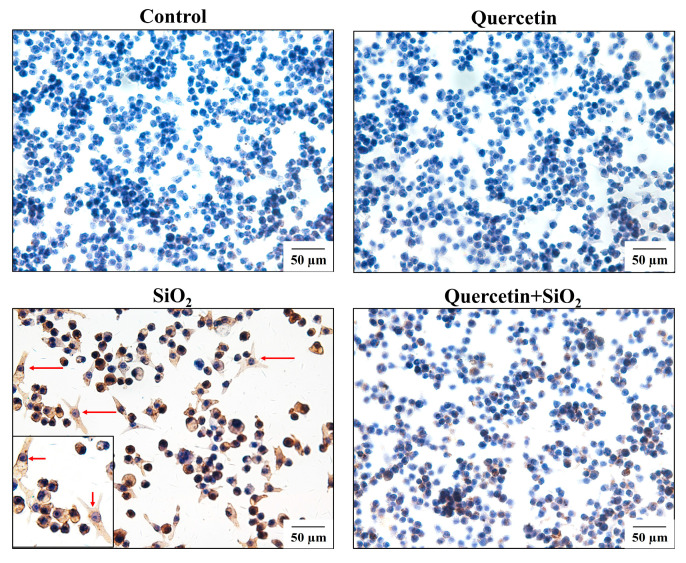
The expression of α-SMA in MH-S, measured by immunocytochemical staining (scale bar = 50 µm).

The MH-S cells were stimulated by SiO_2_ (50 µg/mL) for 24 h to allow macrophages to differentiate into similar myofibroblasts cells with a characteristic spindle shape and cytoplasmic extensions where α-SMA was abundantly present. The cells treated with quercetin were round or oval, with less cytoplasm. The α-SMA was significantly inhibited (Figure 8).

**Figure 9:**
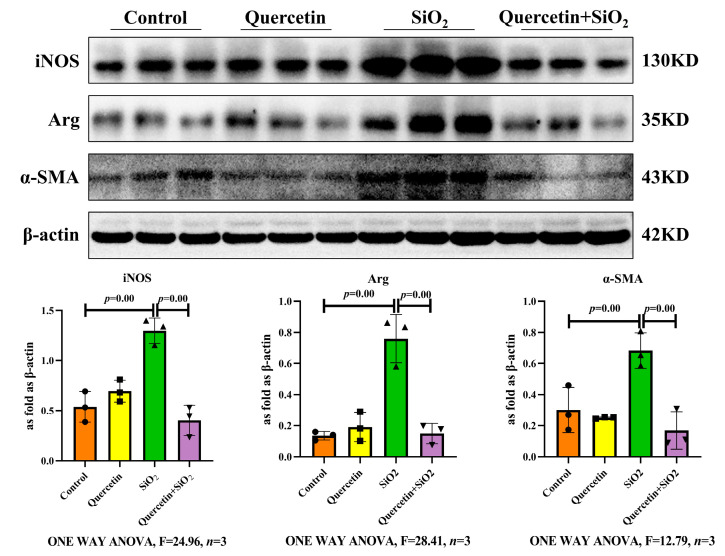
The expressions of iNOS, Arg, and α-SMA in SiO_2_-treated MH-S cells, measured by Western blot. Data are presented as the mean ± SD, *n* = 3 per group.

The expressions of iNOS, Arg, and α-SMA were also validated by Western blot, which showed increased protein levels in the SiO_2_ group. After the quercetin treatment, the protein expressions of iNOS, Arg, and α-SMA caused a significant decrease (Figure 9). These results suggested that quercetin inhibited the MMT process in SiO_2_-induced macrophages.

### 3.4. Quercetin Regulated TGF-β-Smad2/3 Pathway

**Figure 10:**
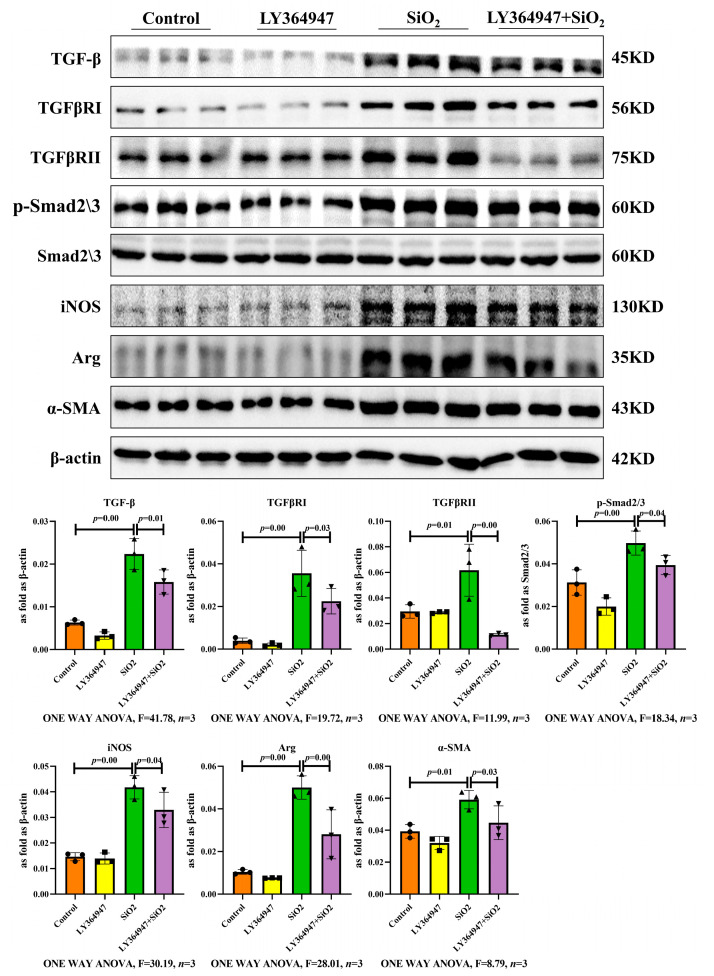
The expressions of TGF-β, TGFβRI, TGFβRII, p-Smad 2/3, iNOS, Arg, and α-SMA in SiO_2_-treated MH-S, measured by Western blot. Data are presented as the mean ± SD, *n* = 3 per group.

**Figure 11:**
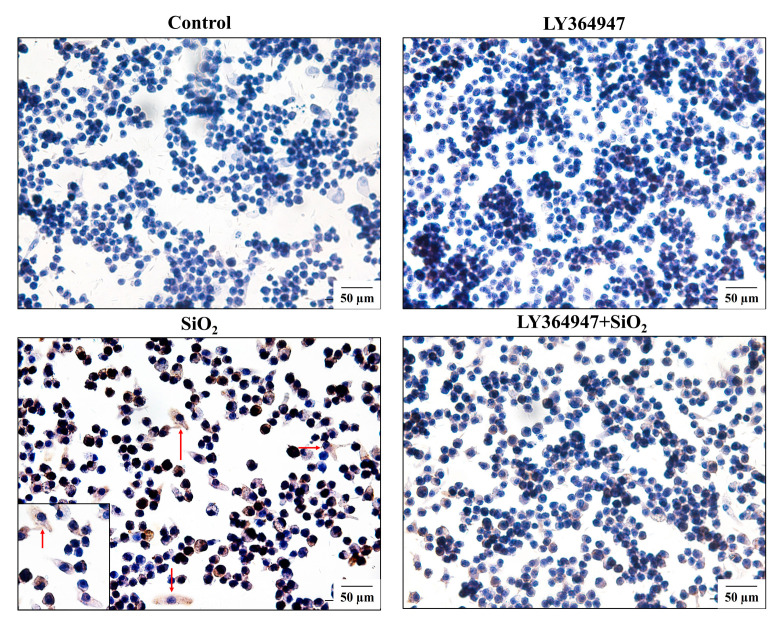
The expression of α-SMA was observed by immunocytochemical staining (scale bar = 50 µm).

As TGF-β-Smad2/3 signaling plays a critical role in fibrosis, we investigated whether it regulates the MMT during silicosis. Western blot results revealed that LY364947, the inhibitor of the TGF-β receptor (TGFβR), led to a decrease in the expressions of TGF-β, TGFβRI, TGFβRII, and phosphorylated Smad 2/3 (p-Smad2/3) in SiO_2_-induced macrophages. Western blot indicated that LY364947 suppressed the expressions of iNOS, Arg, and α-SMA (Figure 10). As shown in Figure 11, LY364947 inhibited the expression of α-SMA compared with the SiO_2_-induced macrophages. These results suggested that the MMT can be regulated by the TGF-β-Smad2/3 signaling pathway.

**Figure 12:**
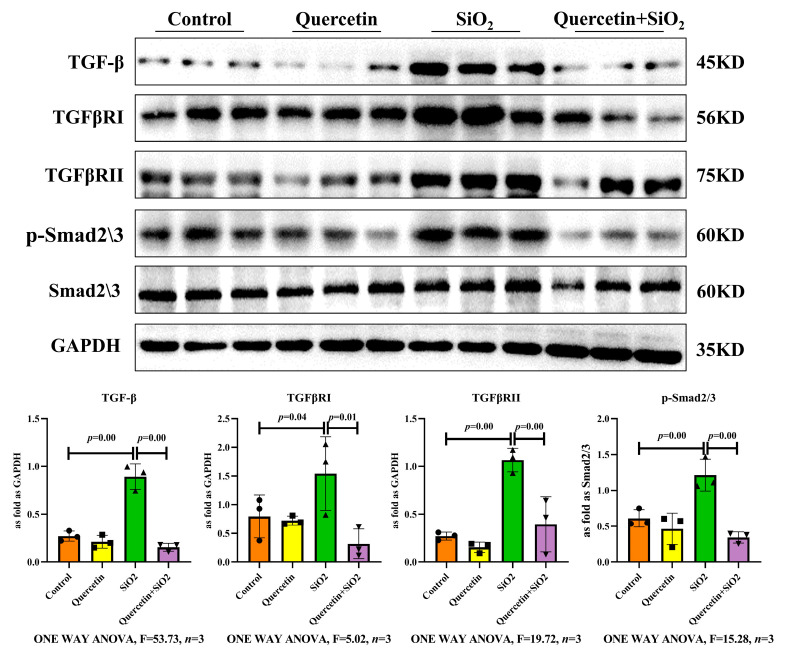
The expressions of TGF-β, TGFβRI, TGFβRII, and p-Smad 2/3 in the lungs, measured by Western blot. Data are presented as the mean ± SD, *n* = 3 per group.

**Figure 13:**
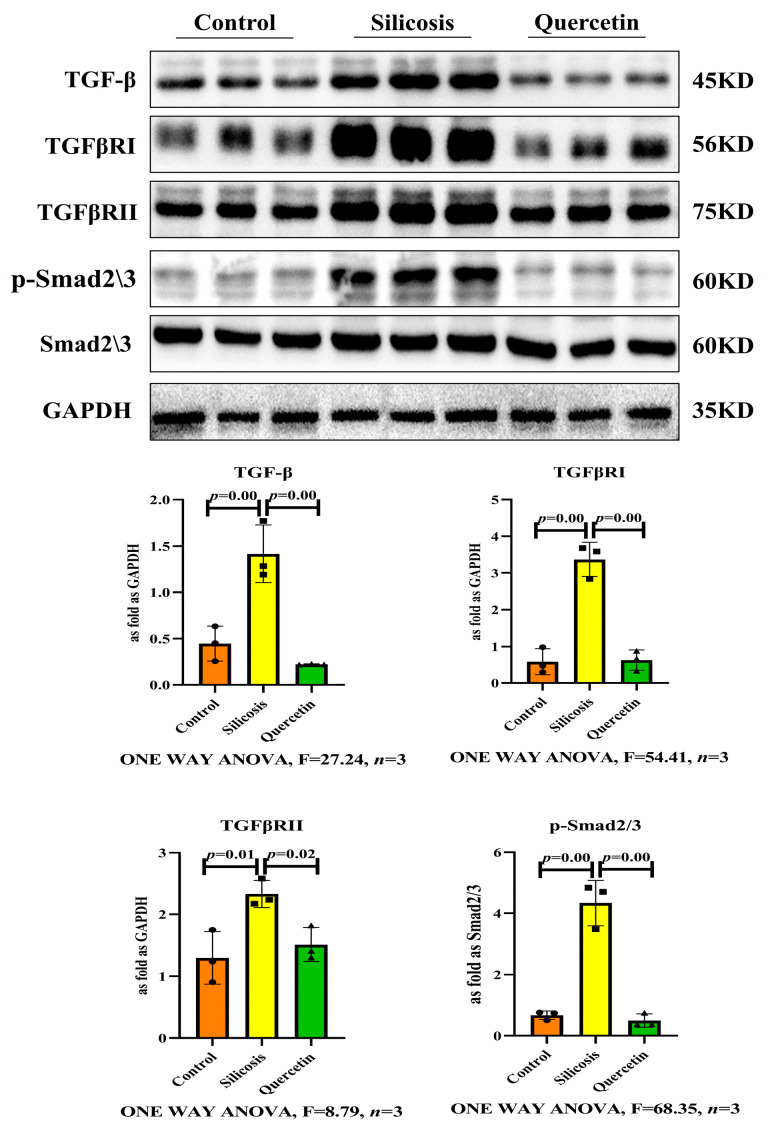
The expressions of TGF-β, TGFβRI, TGFβRII, and p-Smad 2/3 in SiO_2_-treated MH-S, measured by Western blot. Data are presented as the mean ± SD, *n* = 3 per group.

**Figure 14:**
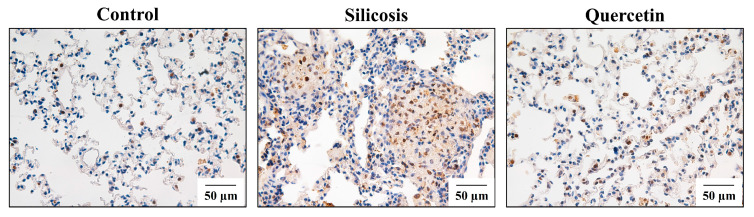
The expression of p-Smad 2/3 in the lung was observed by immunohistochemical staining (scale bar = 50 µm).

Compared with the control group, the expressions of TGF-β, TGFβRI, TGFβRII, and p-Smad 2/3 increased markedly in the SiO_2_ group, and this effect was attenuated by the quercetin treatment (Figure 12). The same changes were observed in the silicotic mice. The protein levels of TGF-β, TGFβRI, TGFβRII, and p-Smad 2/3 were also significantly higher in the silicotic mice and were decreased by quercetin treatment (Figure 13 and Figure 14). These results suggested that quercetin may regulate the TGF-β-Smad2/3 signaling pathway.

## 4. Discussion

Our study evaluated the therapeutic effect of quercetin on silicosis in vivo and in vitro. The results indicated that early treatment with quercetin attenuated the process of macrophage transition. We also found that TGF-β-Smad2/3 signaling is a key regulatory target promoting macrophage transition during silicosis. Our work can provide valuable reference for clinical research and the treatment of silicosis.

Silicosis is a progressive and irreversible disease that is considered refractory to most treatments. Silicosis is characterized by fibroblast proliferation and collagen accumulation. However, the exact pathogenesis of silicosis is unclear and there is an urgent need for effective therapy options. In the present study, we use the 28-day silicosis mice model to evaluate the degree of lung injury and fibrosis by using hematoxylin–eosin and van Gieson’s staining, as well as Western blot, and the results showed a formation of cellular nodules and Col deposition in the lungs with silicosis.

Quercetin, one of the most commonly studied dietary flavonoids, has diverse biological properties that may improve mental/physical performance and reduce the risk of infection. Quercetin is ubiquitously present in vegetables, fruit, tea, and wine and has anti-inflammatory, anti-oxidative, and anti-carcinogenic properties that form the basis for potential benefits to overall health and disease resistance [20]. In addition, there are many studies focusing on the protective effect of quercetin against fibrosis [6,7,8,9]. Previous studies have reported that the early and late therapeutic administration of quercetin ameliorated silicosis in vivo [10]. This study was undertaken to explore the early therapeutic effect of quercetin on silicosis in mice and the potential underlying mechanisms. In this study, the results showed that quercetin treatment can alleviate the formation of a cellular nodule and collagen deposition, providing evidence that quercetin attenuated the structure and improved the function of silicosis in mice models.

Fibrotic responses are driven by a tissue injury accompanied by cellular inflammation in the lungs; thus, lung macrophages have been implicated as playing a significant role in the fibrogenic process [21,22]. The different types of macrophages and their roles in fibrosis have attracted significant attention in recent years [23]. Our study showed that the expressions of iNOS and Arg were significantly reduced by quercetin treatment in vivo and in vitro, indicating that quercetin can reduce macrophage polarization during silicosis.

Myofibroblasts are the main effectors of fibrosis through the synthesis of pathogenic collagen. Recent studies showed that important sources of interstitial myofibroblasts originated from macrophages [24]. The MMT plays a key role in the progression of chronic inflammation into pathogenic fibrosis [25,26]. In the present study, we determined the contributing role of macrophages in the pathogenesis of silicosis, with F4/80+ α-SMA+ MMT cells accounting for a proportion of the myofibroblast population. Co-staining data further elucidated that quercetin effectively attenuated the double-positive percent of CD206 and α-SMA, preventing the accumulation of myofibroblasts in silicosis, which is consistent with the study of MMT cells in the kidney. The Western blot results further confirmed the inhibitory effect of quercetin in the MMT in vitro. Thus, these data suggested that quercetin alleviated silicosis through the MMT in vivo and in vitro.

TGF-β-Smad signaling is a pivotal pathway in fibrosis. TGF-β is a key profibrotic growth factor, mainly generated by macrophages. TGF-β is responsible for fibroblast activation into myofibroblasts and promotes the synthesis of collagen via Smad proteins, a crucial pathway in fibrogenesis [27]. Quercetin effectively inhibited the TGF-β/Smad signaling pathway by promoting the expressions of antifibrogenic genes such as Smad6 and Smad7 while inhibiting the expressions of profibrogenic genes such as Col I, TGF-β, Smad3, and α-SMA. Quercetin may activate antifibrogenic and anti-inflammatory signaling pathways to inhibit the formation of an adenine-induced model of chronic kidney disease [28]. The study demonstrated that tripartite motif-containing 33 (TRIM33) is overexpressed in the lung during fibrotic conditions and plays a protective role against fibrogenesis by inhibiting the TGF-β1 pathway independently of inflammation. The complex interactions between TRIM33, Smad4, and the small heat shock protein B5 (HSPB5) may represent key targets in the prevention of the progression of fibrosis in cases of induced lung fibrosis, as in iatrogenic diseases or in idiopathic pulmonary fibrosis [29]. However, little is known regarding the potential role of TGF-β-Smad signaling in developing the MMT in silicosis. The over-expression of TGF-β, TGFβRI, TGFβRII, and p-Smad 2/3 in the lungs of silicosis mice and macrophages induced by SiO_2_ confirmed the activation of the TGF-β-Smad2/3 signaling pathway. In the present study, we found that SiO_2_ stimulates macrophages to express higher levels of iNOS, Arg, and α-SMA, markers for myofibroblasts, indicating an enhanced MMT, and that the TGFβR inhibitor LY364947 significantly inhibits macrophage polarization and the MMT, showing the regulatory role of the TGF-β-Smad2/3 signaling pathway in the MMT. In addition, quercetin treatment could inhibit the MMT and TGF-β-Smad2/3 signaling.

Here, we found that the mechanism of quercetin is, at least in part, responsible for reducing macrophage polarization and the accumulation of myofibroblasts in the silicosis. On the other hand, the involvement of macrophages in the MMT process and the specific molecular mechanism needs to be further verified, such as extracting lung macrophages, the absence of which is also a limitation of this study. In summary, our study indicated an important benefit of quercetin treatment in silicosis. Moreover, we suggest that the mechanisms of this protection probably operate by preventing both macrophage polarization and the MMT process of macrophages.

## 5. Conclusions

In summary, we suggest that macrophage polarization and the MMT process may be important targets for silicosis, regulated by TGF-β-Smad2/3 signaling. Treatments with quercetin might exert an anti-silicotic effect by inhibiting the MMT and regulating TGF-β-Smad2/3 signaling. In order to apply quercetin to clinical practice, human trials should be included in subsequent studies.

## Data Availability

Not applicable.
